# The development of a consensus-based curriculum for a Bespoke Online Neonatal Education for Transfers (BONNETs) course

**DOI:** 10.1186/s12909-026-09377-3

**Published:** 2026-06-15

**Authors:** Shakti Pillay, Jessica Head, Alan Horn, Wardi de Wet, Ruth Davidge, Maxine Dickson-Hall, Grant Felix, Gugu Kali, Waseela Khan, Bradley Klein, Firdose Nakwa, Maryna Venter, Neville Vlok, Martie Wege, Willem Stassen

**Affiliations:** 1 Department of Paediatrics and Child Health, Division of Neonatology, Faculty of Health Sciences,Groote Schuur Hospital, University of Cape Town, Main Rd Observatory, Cape Town, 7925 South Africa; 2 Department of Family, Community, and Emergency Care, Division of Emergency Medicine, University of Cape Town, Cape Town, South Africa; 3 Critical Care Retrieval And Aeromedical Services, ER24, Cape Town, South Africa; 4 Neonatal Nurses Association of South Africa, Pietermaritzburg, South Africa; 5 Western Cape Department of Health and Wellness, Emergency Medical Services, Cape Town, South Africa; 6 Department of Paediatrics and Child Health, Division of Neonatology, Stellenbosch University, Cape Town, South Africa; 7 Department of Paediatrics and Child Health, School of Clinical Medicine, Chris Hani Baragwanath Academic Hospital, University of the Witwatersrand, Johannesburg, South Africa; 8 Department of Paediatrics and Child Health, Division of Paediatric Intensive Care, Greys Hospital, University of KwaZulu Natal, Pietermaritzburg, South Africa

**Keywords:** “Medical Education”, “Neonatology”, “Patient Transfer”

## Abstract

**Background:**

High neonatal mortality in Sub-Saharan Africa (SSA) is driven by multiple systemic barriers, including insufficient neonatal transport infrastructure and limited provider training. Although specially trained neonatal retrieval teams are recommended for interfacility neonatal transfers, resource constraints lead to non-specialist emergency medical services (EMS) cadres performing these roles, potentially compromising the safe transport of critically ill neonates.

**Objective:**

To develop a bespoke, online neonatal interfacility transfer curriculum, informed by expert consensus and South African research to address critical gaps in provider knowledge and confidence, thereby improving neonatal transfer outcomes.

**Methods:**

Building on Kern’s Six-Step Framework for curriculum development, an initial curriculum was derived through a comprehensive literature review, a retrospective chart analysis of neonatal cases in South Africa, and detailed interviews with experts and learners. Consequently, consensus on this curriculum was sought using a virtual, modified Nominal Group Technique (NGT) over two consensus rounds. A refined course curriculum is proposed.

**Results:**

Fourteen expert participants (neonatologists, paediatricians, neonatal nurses, and Advanced Life Support (ALS) providers with ≥ 3 years of experience) allocated potential outcomes to *Core*, *Extended*, or *Advanced* tiers based on EMS scope of practice. A 75% consensus threshold was applied. Eleven experts completed both rounds (21% attrition). Most were ALS providers (43%), largely employed in the public sector (71%). The final curriculum consists of three sequential courses (Core, Extended, Advanced) which scaffolds learning. The Core course consists of five modules, with each curriculum item achieving > 90% agreement on foundational skills including basic assessment, recognition of critical instability, and escalation pathways. This open-access online course is tailored to resource-limited settings.

**Conclusion:**

A consensus-driven neonatal interfacility transfer curriculum for South Africa was successfully developed, providing a tiered, evidence-based approach to reinforce provider knowledge and confidence. By leveraging expanding internet accessibility, the *Bespoke Online NeoNatal Education for Transfers (BONNETs)* framework mitigates geographic disparities while integrating best practices for safe neonatal transfer. However, rigorous validation across diverse contexts and attention to broader systemic challenges, is essential to achieving sustained improvements. Future research should assess the curriculum’s performance in improving knowledge and confidence, as well as long-term clinical impact on neonatal outcomes.

**Supplementary Information:**

The online version contains supplementary material available at 10.1186/s12909-026-09377-3.

## Background

 Sub-Saharan Africa (SSA) has the highest neonatal mortality rate worldwide at 27 per 1 000 live births [1]. Early neonatal mortality (ENM), defined as deaths within the first seven days of life, remains disproportionately high in this region, with a recent systematic review and meta-analysis estimating a pooled ENM rate of 80.3 per 1 000 live births (95% CI: 66–94.6) [1]. A significant contributor to this mortality burden is the absence of robust neonatal transfer systems, which can result in unsafe transfer practices [2]. Even in regions where neonatal transport services are formally established, their efficacy is frequently compromised by shortages of adequately trained personnel and critical transport equipment [2]. 

In South Africa (SA), the neonatal mortality rate (NMR) has remained relatively static over the past decade at 11–12 per 1 000 live births, reflecting systemic challenges in providing equitable care for critically ill babies [3]. Health resources, including neonatal critical care beds, are unequally distributed, with the majority of beds concentrated in major urban centres across three of the nine provinces (Gauteng, KwaZulu-Natal, and the Western Cape). Consequently, referring hospitals are frequently situated more than 200 km from specialised tertiary centres, creating significant logistical and clinical challenges. In addition, the public healthcare sector, which serves 84% of the population, is markedly under-resourced compared to the private for-profit sector, which serves 16% of the population [4]. This disparity is reflected in the differential outcomes of very low birth weight (VLBW) babies with survival rates of 84.4% in the private sector compared to 70.4% in the public sector [5]. Inadequate neonatal interfacility transfer systems, are among the top ten preventable contributors to neonatal mortality in the country [3]. 

Within SA, neonatal interfacility transfers fall under the mandate of Emergency Medical Services (EMS) with the recommendation that they are conducted by Advanced Life Support (ALS) providers [6]. However, resource constraints often necessitate the involvement of other EMS cadres for emergency transfers of critically ill neonates to higher levels-of-care and the transport of stable neonates between tertiary and step-down healthcare facilities. Two South African studies have highlighted significant deficits in neonatal care knowledge among ALS providers, undermining provider confidence and potentially compromising clinical care during transfers [7, 8]. In one study, 43% of ALS providers felt inadequately prepared for neonatal transfers, with over half reporting no formal neonatal care training despite having 10–30 years of postgraduate experience [7]. As a result, many providers perceive themselves as the “weakest link” in the continuum of care for critically ill neonates [8]. 

The risks associated with neonatal transfers conducted by non-specialised transport teams are well-described in South African studies, with adverse events occurring in more than 75% of these transfers [9–12]. Outborn neonates transferred to tertiary centres have worse outcomes compared to neonates delivered in well-equipped tertiary facilities [13]. This could be ascribed to the development of physiologic instability due to the stressors of transport [14, 15]. In contrast, transport by specialised neonatal teams has been shown to significantly reduce physiological instability and associated complications, improving survival rates from 89% to 96% [15]. 

These findings collectively highlight the urgent need for targeted training programmes in neonatal interfacility transfer. However, EMS providers in SA face multiple barriers to professional development, which includes limited funding, long travel distances to higher education institutions, and insufficient training opportunities [16]. Online, open-access, self-directed learning has emerged as a promising strategy to address these barriers. The utility of this teaching strategy has demonstrated improvements in guideline adherence to traumatic brain injury guidelines among EMS providers in South Africa [17] and enhanced neonatal transfer practices leading to reduced morbidity and mortality outcomes in Mexico [18]. 

Informed by these convergent findings, this study aimed to develop a bespoke online neonatal interfacility transfer curriculum through expert consensus and grounded in prior South African research, with the ultimate goal of improving neonatal transport outcomes [19]. 

## Methods

### Design

A virtual expert stakeholder and consensus meeting was conducted using a modified Nominal Group Technique (NGT) approach to develop the course curriculum. NGT is a structured methodology designed to achieve consensus on a specific topic, facilitating systematic idea generation and problem-solving through inclusive and equitable participation [20]. This study forms the first part of the *Bespoke Online NeoNatal Education for Transfers* (BONNETs) initiative which aims to develop, deliver, and evaluate the impact of an online neonatal interfacility transfer curriculum.

### Ethical considerations

Ethical approval was granted by the University of Cape Town (UCT) Faculty of Health Sciences Human Research Ethics Committee (HREC 591/2022). In the absence of specified reporting guidelines for a NGT, this manuscript adheres to the ACCORD (Accurate Consensus Reporting Document) guidelines [21]. Participation was entirely voluntary, and all data were collected, stored, and analysed in a manner preserving participant anonymity and confidentiality.

### Research paradigm and curriculum development framework

The pedagogical foundation of this project is based on social constructivism and the concept of Communities of Practice (CoP), which emphasises a common goal, active collaboration and the mutual exchange of knowledge, resources and tools [22]. By fostering open dialogue, incorporating diverse perspectives, and encouraging multi-tiered engagement, the CoP framework creates an environment conducive to sustainable capacity-building and long-term collaboration. Notably, the CoP framework is well-aligned with the NGT, enhancing its utility for structured expert consensus.

Building on Kern’s Six-Step Framework for curriculum development, the initial neonatal critical care retrieval curriculum was formulated through a robust methodological process which encompassed a comprehensive literature review, a retrospective chart analysis of neonatal interfacility transfer cases in SA, and detailed interviews with experts and learners [23]. Kern’s framework, was used to translate identified practice gaps into clearly defined educational objectives and to guide the derivation and organisation of draft course-level outcomes. The NGT subsequently served as the validated consensus method to refine, prioritise, and allocate these outcomes across provider levels.

Stakeholder engagement occurred across multiple phases of the project, including the initial needs assessment, expert and learner interviews, EMS provider input, and the NGT consensus process [19]. Following completion of the needs assessment phase, the study team developed an initial set of 13 draft course-level learning outcomes, intended as high-level outcome statements to guide subsequent expert consensus [19]. 

The findings from this foundational work, representing both general and targeted learning needs assessments, are reported in a separate publication [19]. The broader project is guided by the ADDIE (Analyse, Design, Develop, Implement, and Evaluate) model, which provides a systematic and iterative structure for instructional design, curriculum implementation, and subsequent evaluation [24, 25].

### Recruitment and selection

Phase 1 of the study aimed to recruit a minimum of six participants. This threshold was informed by established methodological guidance for the NGT, which recommends expert panels of between five and nine participants to optimise idea generation, promote equitable participation, and maintain manageable group dynamics [20]. 

An expert panel was assembled, consisting of neonatal care providers and critical care retrieval clinicians, who were invited to participate in an online NGT session. Eligible participants included paediatricians, neonatologists, registered nurses working in neonatal intensive care units (NICUs), and ALS providers actively involved in neonatal and critical care retrieval or interfacility transfers. To maintain the requisite level of expertise, all participants were required to have at least three years of professional experience.

Potential participants were identified through relevant professional organisations which included Paediatric Emergency Care South Africa (PECSA), the Emergency Care Society of South Africa (ECSSA), and the United South African Neonatal Association (USANA), in addition to purposive contacts known to the research team.

One month before the scheduled NGT panel discussion, prospective participants received email invitations to participate in a three-hour virtual panel discussion to finalise the curriculum. The email included a comprehensive overview of the study’s objectives, an information sheet on the BONNETs project, and an informed consent form. To facilitate informed participation, all invitees were offered an information-sharing session, either in person or online, prior to providing formal consent.

The information-sharing session was informed by outputs from the preceding needs assessment phase, including findings from the literature review, retrospective analysis of neonatal interfacility transfer cases in South Africa [26], and qualitative input from expert and learner interviews [19]. These materials were synthesised by the study team into a briefing document and draft course-level outcomes to ensure that panel members entered the consensus process with a shared understanding of the project scope and evidence base.

### Procedure

Participants meeting the inclusion criteria were required to confirm their participation by providing written informed consent. Two weeks prior to the panel discussion, participants received the draft course outcomes for the neonatal critical care retrieval curriculum [19] with an explanation of relevant provider qualifications and scopes of practice [6]. 

The NGT panel discussion was hosted virtually using Microsoft Teams (Microsoft Corporation, Washington, United Stated of America), with participant consent for recording and subsequent analysis. Demographic data were collected using Google Forms (Google LLC, California, United States of America). A modified NGT protocol (Table 1) was implemented to ensure a systematic and rigorous approach to idea generation and consensus-building.

**Table 1:** Nominal Group Technique (NGT) Protocol and Procedures

Steps of Data Collection	Description
1. Silent Generation	Panellists had 10 min to reflect individually on each draft outcome. This time frame was considered sufficient as participants had received draft outcomes and supplementary materials two weeks in advance, allowing adequate preparation.
2. Round Robin Sharing	Each of the 13 draft outcomes were addressed sequentially, with participants contributing their ideas until data saturation—defined as informational redundancy—was achieved [20, 26]. Suggestions included adapting outcomes for broader neonatal transfer contexts (beyond critical care retrieval) and proposing specific course content. All contributions were documented and updated in real time, to ensure transparency and shared understanding.
3. Clarification, Discussion and Synthesis	A structured group discussion followed, during which participants synthesised, clarified, and refined their input. Outcomes and content were modified, removed, or reclassified in response to collective feedback and new insights.
4. Consensus Voting and Allocation to Scopes of Practice	Following the virtual meeting, an asynchronous voting process was conducted using Google Forms. Participants were instructed to designate each outcome and its associated content to basic, intermediate, and/or advanced provider categories, considering relevant scopes of practice. A consensus threshold of 75% agreement was predefined, consistent with commonly applied thresholds in Nominal Group Technique and Delphi-based consensus studies and was selected to balance methodological rigour with feasibility in a multidisciplinary expert panel [20, 27].
5. Data Analysis and Verification	Descriptive statistics summarised demographic data such as field of work, years of experience, and provincial representation. Frequency distributions and percentages were calculated to present the consensus agreement levels across the different provider categories. Participants reviewed and validated these findings prior to final curriculum development.

### Curriculum derivation and final consensus

After consensus was achieved in Step 2 (Table 1), the senior author (WS) manually verified each provider level’s scope of practice to ensure compliance with current legislative requirements [6]. In conjunction with the discussion points from Steps 2 and 3, this verification process informed further refinement of the course outcomes and associated content, which were then restructured into three sequential courses, each targeting a distinct audience and EMS function. To validate these revisions, a second round of consensus-building was conducted using Google Forms (Google LLC, California, United States of America), during which participants categorised each outcome and its content as *Core*, *Extended*, or *Advanced*, maintaining the 75% consensus threshold. Descriptive analyses, expressed through frequency distributions and the proportion of positive agreement, were performed to ascertain the level of consensus reached.

Learning outcomes and content recommendations were consolidated into modules in collaboration with experienced learning designers (see acknowledgments), ensuring that the final curriculum adhered to established principles of online pedagogy. This approach not only reinforced academic rigour but also optimised the curriculum’s practicality and relevance for diverse prehospital provider contexts.

## Results

### Demographics

Table 2 outlines the demographics of the expert panel. A total of 14 experts participated in the initial NGT discussion, with 11 completing the second consensus round, reflecting an attrition rate of 21%.

**Table 2:** Demographics of the Expert Panel (*N* = 14)

Field Of Work	(*N*; %)
Advanced Life Support EMS Provider	6; 43%
Neonatology	3; 21%
Paediatrics	2; 14%
Emergency Medicine	1; 7%
Neonatal Nursing	1; 7%
Paediatric Critical Care	1; 7%

Years Of Experience	(*N*; %)
3–5 Years	1; 7%
> 5–10 Years	5; 36%
> 10–15 Years	3; 21%
> 15–20 Years	4; 29%
> 20 Years	1; 7%

Sector Of Work	(*N*; %)
Public Health Sector	10; 71%
Private Health Sector	3; 21%
Academia And Research	1; 7%

Provincial Representation	(*N*; %)
Western Cape	9; 64%
Gauteng	3; 21%
KwaZulu Natal	2; 14%

Neonatal Level of Care	(*N*; %)
Clinical Setting	(*N*; %)
Tertiary Hospital (Referral Centre – all in Public Sector) ^1^	6; 43%
District Hospital ^2^	2; 14%
Neonatal Transfer and Retrieval	4; 29%
Non-Clinical Setting	(*N*; %)
Provincial Programme Development	1; 7%
Academia and Research	1; 7%

^**1**^ Tertiary Hospital: Provides specialist and sub-specialist level services in a neonatal intensive care unit and acts as the referral centre for district hospitals

^**2**^ District hospital: Supports primary care clinics and stabilises sick neonates under supervision of a specialist before transfer to a tertiary centre

Of the three participants who did not complete the second consensus round, two were ALS EMS Providers and one was from Neonatal Nursing.

Most participants were ALS providers active in prehospital care and retrieval (*n* = 6; 43%), and were employed within the public healthcare sector (*n* = 10; 71%). Notably, 13 participants (93%) had over a decade of experience in neonatal retrieval or care. Provincial representation was highest from the Western Cape (*n* = 9; 64%), followed by Gauteng (*n* = 3; 21%) and KwaZulu-Natal (*n* = 2; 14%).

The majority of experts who completed both consensus rounds were actively involved in neonatal interfacility transfer and therefore represented end-users of the curriculum. A subset of participants occupied educational, leadership, or governance roles, contributing to curriculum design, oversight, and strategic perspectives.

### Round one consensus results

Table S1 presents the Round 1 consensus results, including competencies and specific topics designated for basic, intermediate, and advanced provider levels. Early consensus was achieved for several core competencies, which includes recognition and stabilisation of ill neonates and mitigation of transfer-related physiological stress.

Through the NGT process, the initial 13 draft course-level outcomes were refined and operationalised through structured discussion and consensus voting into 51 granular outcomes, reported in Supplementary Table S1, informed by input from the participating stakeholder groups.

### Description of courses according to target audience and EMS function

Based on the second round of consensus, the panel determined that learning outcomes should be organised into three sequential courses, each aligned with a specific target audience and EMS function. Table S1 presents the Round 2 consensus results, which are categorised into the following course levels:

1. BONNETs – Core
  - Designed for EMS providers who may transport neonates independently or as part of a team.
  - Emphasises fundamental neonatal care, recognising critically ill neonates, appropriate escalation pathways, and addressing generic interfacility transport considerations.
  - Delivered exclusively online and open access.
2. BONNETs – Extended
  - Intended for EMS providers acting as the senior member of a non-ALS ambulance crew or as the junior assistant of an ALS transport team.
  - Builds on core competencies by expanding knowledge of neonatal anatomy, pathophysiology (including common birth defects), and other clinical conditions.
  - Self-paced and fully online.
3. BONNETs – Advanced
  - For EMS providers responsible for transporting critically ill neonates with complex conditions as the senior practitioner on an ALS or specialised retrieval unit.
  - Covers advanced procedures, diagnostic techniques, specialised monitoring, and pharmacological interventions for critically ill neonates.
  - Offered in a hybrid format, combining online theoretical components with in-person clinical skills training.

### Consensus-based open-access, online course learning outcomes

Table 3 outlines the learning outcomes across five modules of a bespoke, open-access online course for neonatal interfacility transfer, including the specific module-level outcomes. This represents the BONNETs – Core course.

**Table 3:** Modules and Learning Outcomes for a Bespoke Online Neonatal Interfacility Transfer (BONNETs – Core) Course

Module Name	Outcomes
Module 1: Introduction To Interfacility Transfer Services and Systems	1. Describe the components of an effective transfer service. 2. Define what an adverse event is, together with outlining the importance of reporting and monitoring adverse events during transfer. 3. Describe the pathways and processes for clinical escalation and consultation. 4. Outline the considerations for selecting an appropriate receiving facility for neonate. 5. Describe how referral pathways are developed and how a transfer is arranged. 6. Explain the concept of family-centred care and its importance. 7. Detail the process of information gathering at the referring facility to ensure continuity of care. 8. Appreciate the importance of referral documentation together with outlining the important data points to be gathered in the neonatal population. 9. Note the importance of record-keeping and charting during transfer together with demonstrating competence in the completion of transport-related patient documentation. 10. Outline a process for effective and efficient patient handover at the receiving centre, including documentation.
Module 2: Neonatal Physical Assessment	1. Describe a structured approach to neonatal physical assessment after birth. 2. Recall the normal ranges of vital signs in neonates. 3. Apply a structured neonatal assessment prior to transfer. 4. Formulate a “safe to transfer” checklist. 5. Recognise the critically ill or at-risk neonate.
Module 3: General Care and Transport Stress	1. Explain the importance of skin care in neonatal critical care, including the prevention of skin breakdown. 2. Describe the principles and methods of temperature management in neonatal critical care, highlighting the significance of maintaining thermal stability during transport. 3. Discuss the essentials of neonatal oxygenation and the safe management of supplemental oxygen, including the use of pulse oximetry. 4. Assess the potential effects of sound on neonatal patients during transport and implement measures to reduce noise-related stress and discomfort. 5. Illustrate the critical importance of infection prevention and control measures in neonatal critical care. 6. Explain the principles of neonatal feeding and glycaemic control. 7. Discuss the principles and strategies for fluid management in neonatal critical care, including the assessment and maintenance of fluid balance. 8. Explain the concept of inertial forces in neonatal transport and analyse their potential physiological effects on neonatal patients, as well as implement appropriate strategies to mitigate these effects. 9. Describe the impact of motion, movement, and vibration during neonatal transport on the neonate’s physiology, and apply techniques to minimise the associated risks. 10. Outline the correct methods to secure the neonate during transport to ensure the safety and well-being of the neonatal patient. 11. Identify the risk of emesis (vomiting) in neonatal patients during transport and apply appropriate patient positioning and preventive measures to manage this risk effectively. 12. Describe the physiological changes that occur in neonates at different altitudes during transport and apply strategies to mitigate altitude-related risks. 13. Compare and contrast the unique challenges and considerations associated with ground-based, helicopter, and fixed-wing modes of neonatal transport, and formulate transport plans that address specific physiological effects and risks associated with each mode.
Module 4: Theoretical Concepts of Neonatal Emergency Procedures	1. Describe the structured sequence of steps required for neonatal resuscitation. 2. Apply basic neonatal airway management techniques, including positioning, suctioning, and the use of airway adjuncts. 3. Apply principles of emergency airway support in the neonate, including bag-valve-mask ventilation and use of a pressure-controlled T-piece resuscitator. 4. Identify indications for cardiac compressions in the neonate and outline the principles governing their initiation and performance. 5. Advanced practitioners should also be able to: a) Describe the indications for securing a definitive airway in the neonate and outline the theoretical steps involved. b) Describe the indications for emergency thoracocentesis in the neonate and outline the procedural approach. c) Describe the indications for emergency umbilical venous access and outline the procedural steps involved.
Module 5: Preparation And Monitoring of Equipment	1. Describe the principles and operation of neonatal incubators and their role in maintaining a controlled environment for premature infants. 2. Explain the principles of pulse oximetry and its role in continuous monitoring of the neonate. 3. Describe the procedure for performing a heel lance on a neonate for blood sampling and glucose monitoring. 4. Describe the principles and operation of neonatal resuscitators, including gas-driven and manual devices. 5. Describe the principles and function of humidification devices used in neonatal care. 6. Describe the principles and operation of medication infusion devices, including syringe drivers and infusion pumps. 7. Apply strategies to ensure medication safety throughout the process of neonatal interfacility transfer. 8. Describe the procedure for arterial blood sampling for blood gas analysis.

## Discussion

This study successfully developed a bespoke online curriculum for neonatal interfacility transfer, informed by expert consensus. By employing the NGT approach, a diverse array of experts, spanning neonatology, paediatrics, emergency medicine, and pre-hospital care, contributed insights reflective of multiple healthcare sectors and contexts. Notably, early and robust agreement (86–100%) on core competencies, such as recognising unstable neonates, establishing escalation pathways, and mitigating transfer-related stress, underscores their universal relevance across all provider levels. In contrast, advanced competencies, including mechanical ventilation, endotracheal intubation, and the management of complex neonatal conditions, were appropriately restricted to higher-level providers, in accordance with existing legislative scopes of practice and the elevated risk profile of these interventions [6]. 

### Scaffolded curriculum design

Structuring and categorising the curriculum’s learning outcomes into *Core*, *Extended*, and *Advanced* tiers aligns the online, open-access course with national EMS scope-of-practice guidelines, making it broadly applicable to varying clinical roles and levels of expertise [6]. This framework provides a robust, scaffolded approach to training, meeting diverse learner needs and facilitating adaptability across multiple healthcare contexts.

### Bridging gaps in SSA

In many SSA countries, neonatal interfacility transfers lack formal protocols and are frequently undertaken by providers who lack specialised neonatal transport training [2]. Consequently, the BONNETs - Core course offers a promising avenue for bridging critical knowledge gaps in SA and potentially across the wider African region. It can serve as a supplementary resource for providers experienced in neonatal care but unfamiliar with transport procedures or serve as an introductory step toward a career in advanced neonatal retrieval. By leveraging expanding internet access and the success of previous e-learning initiatives [17], the BONNETs - Core course offers a scalable training solution that mitigates geographic disparities and increases accessibility for healthcare providers in both urban and rural settings. While this approach broadens the reach of neonatal transport training, comprehensive validation of the curriculum’s content and contextual suitability remains essential before large-scale implementation for a diverse provider audience. In addition to clinical training gaps, variability in interfacility documentation practices and communication systems across facilities in SSA may pose challenges to the effective integration of standardised transfer curricula, highlighting the need for alignment with local documentation workflows and referral systems.

### Local adaptation and feasibility

Currently, no open-access, online training resources specifically tailored to neonatal interfacility transfers exists within the African context. In response, the BONNETs – Core course was developed through a comprehensive needs assessment involving a heterogenous sample [19], aligning international best practices for safe neonatal transfer with local realities such as resource constraints, geographic disparities, and limited training opportunities. This accessible e-learning approach not only enhances the relevance of the BONNETs – Core course in SA but also holds promise for other African regions aiming to improve provider competencies in neonatal interfacility transfers.

### Towards a systems approach

However, while enhancing provider confidence and competence is a vital first step toward safer neonatal transport, training must occur in parallel with broader systemic strengthening to ensure sustainable impact. Five critical elements need to be addressed: targeted patient populations (neonates), validated case selection criteria, dedicated crews equipped with specialised training, appropriate transport equipment aligned to neonatal needs, and continuous quality management and training [2, 26, 28]. Adopting a rigorous systems approach is vital, given that insufficient interfacility transport infrastructures remains a preventable cause of neonatal mortality and morbidity in critically ill neonates [3]. Integrating robust educational initiatives with systemic enhancements can maximise the impact of interfacility transport interventions and, ultimately, improve neonatal outcomes throughout the African region.

### Implementing ADDIE

Implementing and evaluating the neonatal interfacility transfer curriculum within the ADDIE framework represents the next phase of this project [24, 25]. Future research should extend beyond the assessment of knowledge acquisition and learner confidence to evaluate the impact of the programme on outcomes such as neonatal stability, morbidity, and mortality before, during, and after interfacility transfer. Given the diversity of regional healthcare systems across SSA, further contextual adaptation of course content and learning outcomes may be required, supported by collaboration with local learners and clinical and educational stakeholders.

The combined applications of a needs-driven curriculum development framework (Kern’s Six-Step Framework) [23] with a validated consensus methodology (NGT) introduces a pragmatic and transferable approach to curriculum design, with potential relevance for educators developing context-specific training programmes in resource-constrained or evolving healthcare systems [20]. 

While the BONNETs curriculum is delivered through an online, self-directed learning platform, effective translation of knowledge into clinical practice will require structured supervision and a formalised assessment strategy. The proposed assessment framework incorporates formative assessments and staged summative evaluations to support educational governance, quality assurance, and monitoring of learner progression [19]. Future implementation phases will incorporate refresher training delivered at context-appropriate intervals, supported by site-level quality improvement processes to promote sustained clinical competence and enable longitudinal evaluation of educational and clinical impact.

The goal of this educational intervention is to drive demonstrable improvements in patient care. Robust outcome data will therefore be essential to confirm that the BONNETs curriculum translates into safer and more effective neonatal interfacility transfers across heterogenous resource settings.

### Limitations

While the study sample reflects a diversity of expertise, scope of work, and levels of experience, the provincial representation mirrors regions with the most established neonatal resources and training. This focus may align the curriculum more closely with these better-resourced settings, which could pose challenges when applying the curriculum to regions with differing resource constraints. Despite a relatively small sample size, which could impact the validity of the curriculum items, the study adheres to the recommended minimal requirements for nominal group technique (NGT) studies [20, 27].

Additionally, this sample represents the culmination of a comprehensive process that included stakeholder engagement across multiple phases [19], literature reviews, and analysis of real-world patient data [26]. These steps strengthen the rigour of our findings and ensure the curriculum is grounded in both evidence and practical insights.

## Conclusion

We present a consensus-driven, bespoke curriculum designed for an online neonatal interfacility transfer course. BONNETs provides a tiered, evidence‐based learning pathway intended to address critical gaps in provider knowledge, competence and confidence. By offering robust and accessible online training, this initiative has the potential to bolster neonatal transfer safety and improve survival outcomes, particularly for VLBW babies, while mitigating geographic barriers to training. Future research should explore the curriculum’s impact on clinical outcomes, including its broader applicability to other areas of paediatric emergency care. Further, research should assess its capacity to reduce neonatal mortality and morbidity through the provision of scalable, context-specific e-learning in resource-constrained health systems.

## Supplementary Information

Supplementary Material 1.

## Data Availability

The datasets generated and analysed during the current study are available from the corresponding author on reasonable request.
