# Supplementary material for: The development of a consensus-based curriculum for a Bespoke Online Neonatal Education for Transfers (BONNETs) course
Source: BMC Med Educ. 2026 Jun 15;26:1346. doi: 10.1186/s12909-026-09377-3 (PMC13495191; doi:10.1186/s12909-026-09377-3)
Supplement: Supplementary file 1 [file 12909_2026_9377_MOESM1_ESM.docx]

**THE DEVELOPMENT OF A CONSENSUS-BASED CURRICULUM FOR A BESPOKE ONLINE NEONATAL EDUCATION FOR TRANSFERS (BONNETs) COURSE**

Shakti Pillay, ^1^ Jessica Head, ^2^ Alan Horn, ^1^ Wardi de Wet, ^3^ Ruth Davidge, ^4^ Maxine Dickson-Hall, ^3^ Grant Felix, ^5^ Gugu Kali, ^6^ Waseela Khan, ^2^ Bradley Klein, ^5^ Firdose Nakwa, ^7^ Maryna Venter, ^2,3^ Neville Vlok, ^2^ Martie Wege, ^8^ Willem Stassen ^2^

1. Division of Neonatology; Department of Paediatrics and Child Health; Groote Schuur Hospital, University of Cape Town, South Africa
2. Division of Emergency Medicine; Department of Family, Community, and Emergency Care; University of Cape Town; South Africa
3. Critical care retrieval and aeromedical services; ER24; South Africa
4. Neonatal Nurses Association of South Africa
5. Emergency Medical Services; Western Cape Department of Health and Wellness; South Africa
6. Division of Neonatology; Department of Paediatrics and Child Health; Stellenbosch University, South Africa
7. Department of Paediatrics and Child Health; Chris Hani Baragwanath Academic Hospital; School of Clinical Medicine; University of the Witwatersrand; Johannesburg; South Africa
8. Division of Paediatric intensive care; Department of Paediatrics and Child Health; Greys Hospital; University of Kwazulu Natal; Pietermaritzburg; South Africa

Corresponding Author:	Shakti Pillay

Division of Neonatology

Faculty of Health Sciences

University of Cape Town

Groote Schuur Hospital

Main Rd

Observatory

Cape Town

South Africa

7925

Email: [shakti.pillay@uct.ac.za](mailto:shakti.pillay@uct.ac.za)

**Word count:** 2833

**SUPPLEMENTARY MATERIAL**

| **Table S1** |  | | |  |
| --- | --- | --- | --- | --- |
|  | **Round 1 (n=14)** | | | **Round 2 (n=11)** |
| **Curriculum Elements** | **BLS Agreement**  (n; %) | **ILS Agreement**  (n; %) | **ALS Agreement**  (n; %) | **Included**  n; % |
| **Describe Anatomy and Physiology as it Relates to a Neonate** | | | | |
| Describe transition from intra-uterine to extra-uterine life | 7; 50% | 11; **79%** | 14; **100%** | E: 11; 100% |
| Understand anatomical features of common birth defects | 4; 29% | 7; 50% | 14; **100%** | E: 9; 82%* |
| Provide an understanding of anatomy, physiology, and pathophysiology of important neonatal conditions | 5; 36% | 8; 57% | 14; **100%** | E: 9; 82%* |
| **Components of an Effective Transport Service** | | | | |
| The five components of Critical Care Retrieval Service (CCRS): Specific neonatal populations, case selection, dedicated crew with training, equipment, continuous quality management | 8; 57% | 9; 64% | 13; **93%** | C: 11; 100% |
| Adverse events | 11; **79%** | 13; **92%** | 14; **100%** | C: 11; 100% |
| Selection of appropriate receiving facilities | 10; 71% | 12; **86%** | 14; **100%** | C: 10; 91% |
| Escalation and consultation pathways | 13; **93%** | 14; **100%** | 14; **100%** | C: 11; 100% |
| Recognising instability that is at risk of unsafe transfer | 12; **86%** | 14; **100%** | 14; **100%** | C: 11; 100% |
| Navigating referral pathways | 7; 50% | 11; **79%** | 14; **100%** | C: 9; 82% |
| **Apply Transport Considerations and Effect of Transport Stress on Range of Conditions Specific to the Critical Neonate** | | | | |
| Respiratory distress syndrome | 9; 64% | 13; **93%** | 13; **93%** | A: 11; 100% |
| Congenital heart defects | 1; 7% | 7; 50% | 13; **93%** | A: 11; 100% |
| Prematurity: Handling and viability | 10; 71% | 13; **93%** | 13; **93%** | A: 11; 100% |
| Infection; Sepsis | 8; 57% | 12; **86%** | 13; **93%** | A: 11; 100% |
| Bronchopneumonia | 6; 43% | 9; 64% | 13; **93%** | A: 11; 100% |
| Diaphragmatic hernia | 0; 0% | 2; 14% | 13; **93%** | A: 11; 100% |
| Meconium aspiration | 3; 21% | 8; 57% | 13; **93%** | A: 11; 100% |
| Persistent pulmonary hypertension | 0; 0% | 2; 14% | 13; **93%** | A: 11; 100% |
| Hypoxic ischaemic encephalopathy | 2; 14% | 7; 50% | 13; **93%** | A: 11; 100% |
| Low birthweight | 14; **100%** | 14; **100%** | 13; **93%** | A: 11; 100% |
| Convulsions | 7; 50% | 11; **79%** | 13; **93%** | A: 11; 100% |
| Hydrocephalus | 1; 7% | 5; 36% | 13; **93%** | A: 11; 100% |
| Bowel obstruction | 2; 14% | 6; 43% | 13; **93%** | A: 11; 100% |
| Gastroschisis | 1; 7% | 4; 29% | 13; **93%** | A: 11; 100% |
| Necrotising enterocolitis | 1; 7% | 3; 21% | 13; **93%** | A: 11; 100% |
| Malnutrition and dehydration | 3; 21% | 8; 57% | 12; **86%** | A: 11; 100% |
| Bleeding in the neonate | 5; 36% | 8; 57% | 13; **93%** | A: 11; 100% |
| Macrosomia | 5; 36% | 5; 36% | 13; **93%** | A: 11; 100% |
| Neonates with low APGAR scores | 9; 64% | 11; **79%** | 13; **93%** | A: 11; 100% |
| Hypothermia | 13; **93%** | 14; **100%** | 13; **93%** | A: 11; 100% |
| Hypoglycaemia | 12; **86%** | 13; **93%** | 13; **93%** | A: 11; 100% |
| Jaundice | 11; **79%** | 12; **86%** | 13; **93%** | A: 11; 100% |
| Tracheoesophageal fistula | 0; 0% | 2; 14% | 13; **93%** | A: 11; 100% |
| Shock | 6; 43% | 10; 71% | 13; **93%** | A: 11; 100% |
| Traumatic birth | 9; 64% | 11; **79%** | 13; **93%** | A: 11; 100% |
| Abdominal distention | 7; 50% | 10; 71% | 13; **93%** | A: 11; 100% |
| **Explain Continuity and Maintenance of Neonatal Critical Care and Its Application During Transport** | | | | |
| Fluid management | 3; 21% | 12; **86%** | 14; **100%** | C: 10; 91%* |
| Feeding and glycaemic control | 10; 71% | 13; **93%** | 14; **100%** | C: 11; 100% |
| Skin care | 13; **93%** | 14; **100%** | 14; **100%** | C: 11; 100% |
| Temperature control | 14; **100%** | 14; **100%** | 14; **100%** | C: 11; 100% |
| Oxygen management | 14; **100%** | 14; **100%** | 14; **100%** | C: 11; 100% |
| Hearing protection | 11; **79%** | 12; **86%** | 14; **100%** | C: 11; 100% |
| Infection prevention and control | 13; **93%** | 13; **93%** | 14; **100%** | C: 11; 100% |
| **Describe and Prepare Documentation Required for Safe Transitions of Care During The Handover of a Neonate** | | | | |
| Information gathering at referring facility | 14; **100%** | 14; **100%** | 14; **100%** | C: 11; 100% |
| Referral documentation | 14; **100%** | 14; **100%** | 14; **100%** | C: 10; 91% |
| Handover documentation | 14; **100%** | 14; **100%** | 14; **100%** | C: 11; 100% |
| Record-keeping and charting during transfer | 14; **100%** | 14; **100%** | 14; **100%** | C: 11; 100% |
| **Demonstrate Theoretical Knowledge in Application of Emergency Procedures For Neonatal Resuscitation and Stabilisation** | | | | |
| Endotracheal intubation | 0; 0% | 1; 7% | 14; **100%** | C: 9; 82% * |
| Needle thoracentesis | 0; 0% | 2; 14% | 14; **100%** | C: 9; 82% * |
| Cardiopulmonary resuscitation | 13; **93%** | 14; **100%** | 14; **100%** | C: 10; 91% |
| Intravenous access | 2; 14% | 8; 57% | 14; **100%** | C: 10; 91% * |
| Airway management and positioning | 11; **79%** | 13; **93%** | 14; **100%** | C: 11; 100% |
| Suctioning | 13; **93%** | 14; **100%** | 14; **100%** | C: 11; 100% |
| Bag valve mask ventilation | 14; **100%** | 14; **100%** | 14; **100%** | C: 11; 100% |
| **Demonstrate the Safe Use of Equipment for Monitoring and Management of the Neonate During Transport, Heating, Mechanical Ventilation and Infusions** | | | | |
| Incubators | 12; **86%** | 14; **100%** | 14; **100%** | C: 11; 100% |
| Infusion devices | 2; 14% | 8; 57% | 14; **100%** | C: 10; 91% |
| Mechanical ventilators | 0; 0% | 2; 14% | 14; **100%** | A: 11; 100% |
| Nasal cannula and oxygen masks | 13; **93%** | 14; **100%** | 14; **100%** | C: 11; 100% |
| Humidification devices | 3; 21% | 10; 71% | 14; **100%** | C: 9; 82% * |
| Setup of positive pressure devices | 9; 64% | 11; **79%** | 14; **100%** | C: 9; 82% * |
| Pulse oximetry | 12; **86%** | 14; **100%** | 14; **100%** | C: 11; 100% |
| Point of care sampling (excluding heel prick) | 3; 21% | 6; 43% | 14; **100%** | C: 8; 73% * |
| Heel prick for blood glucose |  |  |  | C: 11; 100% |
| **Describe and Demonstrate Monitoring, Care, Management and Troubleshooting of Indwelling Attachments as they Relate to Neonatal Critical Care And Transport, Including Drains and Different Methods of Vascular Access** | | | | |
| Arterial lines | 0; 0% | 1; 7% | 14; **100%** | A: 11; 100% |
| Central venous access | 0; 0% | 1; 7% | 14; **100%** | A: 11; 100% |
| Intraosseous access | 0; 0% | 6; 43% | 14; **100%** | A: 11; 100% |
| Peripheral venous access | 5; 36% | 12; **86%** | 14; **100%** | A: 11; 100% |
| Umbilical lines | 2; 14% | 7; 50% | 14; **100%** | A: 11; 100% |
| Colostomy care | 3; 21% | 5; 36% | 14; **100%** | A: 11; 100% |
| Variety of drains or catheters | 1; 7% | 5; 36% | 14; **100%** | A: 11; 100% |
| Chest drains | 0; 0% | 2; 14% | 14; **100%** | A: 11; 100% |
| Gastric tubes | 7; 50% | 12; **86%** | 14; **100%** | A: 11; 100% |
| Endotracheal tube | 0; 0% | 3; 21% | 14; **100%** | A: 11; 100% |
| **Describe and Demonstrate the Monitoring, Care and Management of a Variety of Medications as they Relate to Neonatal Critical Care and Transport** | | | | |
| Fluid maintenance | 4; 29% | 12; **86%** | 14; **100%** | C: 11; 100% * |
| Sedatives and sedation | 0; 0% | 5; 36% | 14; **100%** | A: 11; 100% |
| Ionotropic and vasoactive medications | 0; 0% | 2; 14% | 14; **100%** | A: 11; 100% |
| Analgesics | 2; 14% | 5; 36% | 14; **100%** | A: 11; 100% |
| Antibiotics | 4; 29% | 7; 50% | 14; **100%** | A: 11; 100% |
| Prostaglandins | 1; 7% | 5; 36% | 14; **100%** | A: 11; 100% |
| Heparin | 0; 0% | 3; 21% | 14; **100%** | A: 11; 100% |
| Sodium bicarbonate | 1; 7% | 4; 29% | 14; **100%** | A: 11; 100% |
| Dextrose including oral glucose | 8; 57% | 13; **93%** | 14; **100%** | A: 11; 100% |
| Anti-convulsant medication | 2; 14% | 7; 50% | 14; **100%** | A: 11; 100% |
| Vitamin K | 4; 29% | 8; 57% | 14; **100%** | A: 11; 100% |
| Caffeine | 5; 36% | 7; 50% | 14; **100%** | A: 11; 100% |
| **Demonstrate the Initial and Ongoing Assessment of a Neonate During Neonatal Critical Care and Transport** | | | | |
| Recognising the ill neonate | 14; **100%** | 14; **100%** | 14; **100%** | C: 11; 100% |
| Neonatal assessment | 13; **93%** | 14; **100%** | 14; **100%** | C: 11; 100% |
| Normal vital sign ranges, term and preterm | 14; **100%** | 14; **100%** | 14; **100%** | C: 11; 100% |
| **Apply and Analyse a Variety of Methods Used for the Monitoring of a Critically-Ill Neonate during Transfer** | | | | |
| Electrocardiography | 1; 7% | 8; 57% | 14; **100%** | A: 11; 100% |
| Pulse oximetry | 12; **86%** | 14; **100%** | 14; **100%** | C: 11; 100% |
| Capnography | 0; 0% | 2; 14% | 14; **100%** | A: 11; 100% |
| Arterial blood gas analysis | 0; 0% | 2; 14% | 14; **100%** | A: 11; 100% |
| Fluid balance | 2; 14% | 9; 64% | 14; **100%** | A: 11; 100% |
| Glucose management | 7; 50% | 11; **79%** | 14; **100%** | C: 11; 100% |
| Perfusion, including peripheral | 8; 57% | 10; 71% | 14; **100%** | C: 11; 100% |
| Invasive and non-invasive blood pressure | 2; 14% | 8; 57% | 14; **100%** | A: 11; 100% |
| Thermoregulation | 14; **100%** | 14; **100%** | 14; **100%** | C: 11; 100% |
| **Describe the Physiological Effects the Different Modes of Transport can have on the Neonate and Apply Methods to Mitigate Potential Risks During Transport** | | | | |
| Considerations for ground-based, helicopter and fixed-wing modes of transport | 8; 57% | 12; **86%** | 14; **100%** | A: 9; 82% |
| Inertial forces | 7; 50% | 8; 57% | 14; **100%** | C: 8; 73% |
| Motion, movement, vibration | 11; **79%** | 12; **86%** | 14; **100%** | C: 11; 100% |
| Sound | 11; **79%** | 13; **93%** | 14; **100%** | C: 11; 100% |
| Patient packaging, with considerations for Kangaroo Mother Care (pending evidence) | 13; **93%** | 12; **86%** | 12; **86%** | C: 11; 100% |
| Family-centred care, and the mother | 14; **100%** | 14; **100%** | 14; **100%** | C: 11; 100% |
| Emesis risk | 10; 71% | 11; **79%** | 14; **100%** | C: 9; 82% * |
| Altitude and flight physiology | 1; 7% | 6; 43% | 14; **100%** | C: 8; 73% * |
| **Describe and Demonstrate the Monitoring, Care and Management of a Variety of Ventilation Modes and Methods as they Relate to Neonatal Critical Care and Transport** | | | | |
| Bag valve mask ventilation | 14; **100%** | 14; **100%** | 14; **100%** | C: 11; 100% |
| Continuous Positive Pressure Ventilation (CPAP) | 0; 0% | 7; 50% | 14; **100%** | A: 11; 100% |
| Heated circuits | 0; 0% | 5; 36% | 14; **100%** | A: 11; 100% |
| Humidification | 4; 29% | 9; 64% | 14; **100%** | A: 11; 100% |
| T-piece resuscitator | 10; 71% | 12; **86%** | 14; **100%** | C: 9; 82% * |
| Oxygen Blending | 8; 57% | 11; **79** | 14; **100%** | A: 11; 100% |
| Oscillation (basic knowledge) | 0; 0% | 0; 0% | 14; **100%** | A: 11; 100% |
| Mechanical ventilation | 0; 0% | 2; 14% | 14; **100%** | A: 11; 100% |
| High Flow Oxygen | 3; 21% | 7; 50% | 14; **100%** | A: 11; 100% |
| C= Core  E= Extended  A= Advanced  *Include in curriculum, but only for advanced provider | | | | |
